# Effectiveness of BNT162b2 XBB Vaccine Against XBB and JN.1 Sublineages

**DOI:** 10.1093/ofid/ofae370

**Published:** 2024-07-01

**Authors:** Sara Y Tartof, Jeff M Slezak, Laura Puzniak, Timothy B Frankland, Bradley K Ackerson, Luis Jodar, John M McLaughlin

**Affiliations:** Department of Research and Evaluation, Kaiser Permanente Southern California, Pasadena, California, USA; Department of Research and Evaluation, Kaiser Permanente Southern California, Pasadena, California, USA; Global Vaccines and Antivirals, Pfizer Inc, Collegeville, Pennsylvania, USA; Center for Integrated Health Care Research (CIHR), Kaiser Permanente Hawaii, Honolulu, Hawaii, USA; Department of Pediatric Infectious Diseases, Southern California Permanente Medical Group, Harbor City, California, USA; Global Vaccines and Antivirals, Pfizer Inc, Collegeville, Pennsylvania, USA; Global Vaccines and Antivirals, Pfizer Inc, Collegeville, Pennsylvania, USA

**Keywords:** COVID-19, vaccine effectiveness, JN.1, XBB, BNT162b2

## Abstract

We provide updated results (11 October 2023 through 29 February 2024) from our previously conducted test-negative case-control study in Kaiser Permanente Southern California to evaluate sublineage-specific effectiveness of the BNT162b2 XBB1.5-adapted vaccine. Results suggest that XBB1.5-adapted vaccines may have reduced effectiveness against JN.1 versus XBB sublineages.

JN.1, a phylogenetically and antigenically distinct sublineage from XBB, was first identified in August 2023 and became the predominant severe acute respiratory syndrome coronavirus 2 (SARS-CoV-2) sublineage globally by the end of December 2023 [1]. Despite early neutralization studies showing signs of potential immune evasion [2, 3], only a few studies in isolated populations [4–6] have assessed real-world effectiveness of XBB1.5-adapted coronavirus disease 2019 (COVID-19) vaccines against JN.1-related endpoints. We assessed effectiveness of BNT162b2 XBB1.5-adapted vaccine (Pfizer-BioNTech 2023–2024 formulation; hereafter referred to as BNT162b2 XBB vaccine) against XBB and JN.1 sublineages in a large, diverse healthcare system in the United States (US).

## METHODS

We updated our previous test-negative case-control BNT162b2 XBB vaccine effectiveness (VE) analysis [7] to (1) include a longer study period (10 October 2023 through 29 February 2024 [instead of 10 December 2023]) to allow for additional JN.1 cases, and (2) stratify VE estimates by time since vaccination and SARS-CoV-2 sublineage (ie, likely XBB- or JN.1-related). Otherwise, selection criteria were identical to our prior analysis [7]. In brief, patients were ≥18 years of age who were diagnosed with acute respiratory infection (Supplementary Table 1) and tested for SARS-CoV-2 using polymerase chain reaction (PCR) during a hospital admission or emergency department (ED) or urgent care (UC) encounter at Kaiser Permanente Southern California during the study period. Cases were those with a positive SARS-CoV-2 PCR test; controls tested negative and had no encounters, in any setting, with a positive SARS-CoV-2 test in the prior 90 days. Likely SARS-CoV-2 strain was determined based on a hierarchy of available information including (1) whole genome sequencing (WGS), (2) spike gene target failure (SGTF), or (3) periods of >80% sublineage predominance based on WGS data from US Health and Human Services Region 9 [1]. Odds of receipt of a BNT162b2 XBB vaccine were compared to the odds of not receiving XBB vaccine of any kind (including previously vaccinated and unvaccinated persons) across cases and controls. Adjusted odds ratios (ORs) and 95% confidence intervals (CIs) were calculated using logistic regression and SAS version 9.4. VE was calculated as 1 − OR multiplied by 100% (Supplementary Appendix).

## RESULTS

Of 59 058 acute respiratory infection encounters meeting eligibility criteria, 52 036 met selection criteria (8732 [16.8%] hospital admissions, 43 304 [83.2%] ED/UC encounters; Supplementary Figure 1). Median age was 56 years (interquartile range, 38–72 years); Supplementary Tables 2 and 3 describe participant characteristics by case-control and exposure status, respectively. Overall, 7572 of 52 036 (14.6%) tested SARS-CoV-2 positive, of which 2475 (32.7%) and 2209 (29.2%) were confirmed as likely XBB and JN.1 sublineages, respectively. In total, 6923 of 52 036 (13.3%) received BNT162b2 XBB vaccine with median time since vaccination of 58 days (range, 15–156 days). Of 7572 cases and 44 464 controls, 753 (9.9%) and 6170 (13.9%), respectively, received BNT162b2 XBB vaccine.

**Figure 1.**
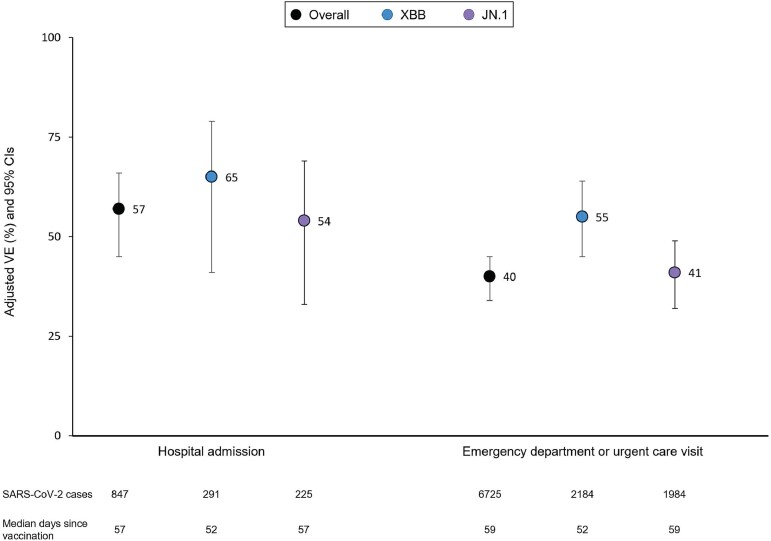
Effectiveness of BNT162b2 XBB vaccine by coronavirus disease 2019 encounter type and severe acute respiratory syndrome coronavirus 2 (SARS-CoV-2) sublineage—10 October 2023 through 29 February 2024. Models adjusted for week of encounter, age, sex, self-reported race/ethnicity, body mass index, Charlson Comorbidity Index, prior SARS-CoV-2 infection, and utilization history (flu and pneumococcal vaccination, inpatient, emergency department, and outpatient encounters in prior year). Abbreviations: CI, confidence interval; SARS-CoV-2, severe acute respiratory syndrome coronavirus 2; VE, vaccine effectiveness.

Overall (including all sublineages) adjusted BNT162b2 XBB VE was 57% (95% CI, 45%–66%) against COVID-19–related hospital admission and 40% (95% CI, 34%–45%) against ED/UC visits. Against XBB sublineages, VE was 65% (95% CI, 41%–79%) for hospitalization and 55% (95% CI, 45%–64%) for ED/UC, compared to 54% (95% CI, 33%–69%) and 41% (95% CI, 32%–49%) against JN.1 sublineages, respectively (Figure 1).

**Figure 2.**
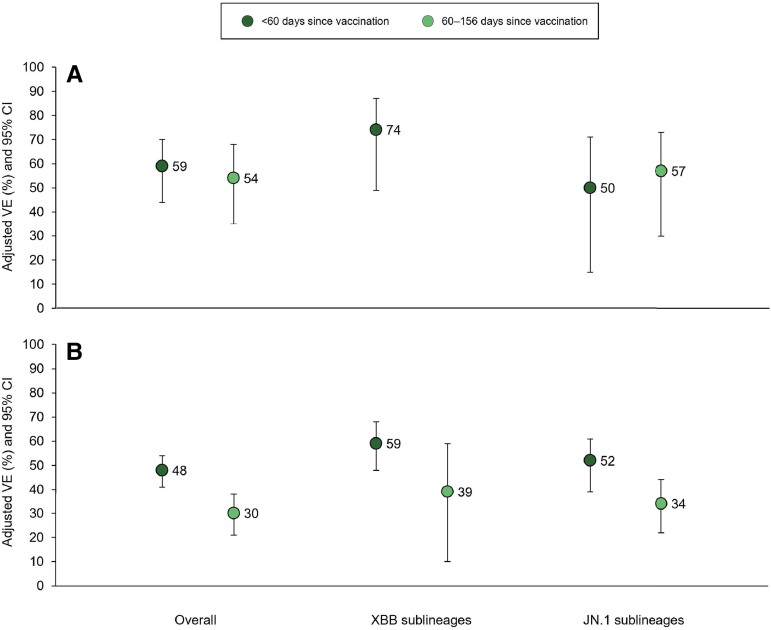
Effectiveness of BNT162b2 XBB vaccine by coronavirus disease 2019 encounter type, time since vaccination, and severe acute respiratory syndrome coronavirus 2 (SARS-CoV-2) sublineage—10 October 2023 through 29 February 2024. *A*, Effectiveness against hospital admission. *B*, Effectiveness against emergency department (ED)/urgent care visits. Vaccine effectiveness (VE) ≥2 months after vaccination could not be calculated for XBB sublineages due to small sample size. Models adjusted for week of encounter, age, sex, self-reported race/ethnicity, body mass index, Charlson Comorbidity Index, prior SARS-CoV-2 infection, and utilization history (flu and pneumococcal vaccination, inpatient, ED, and outpatient encounters in prior year). Abbreviations: CI, confidence interval; VE, vaccine effectiveness.

When stratified by time since vaccination, VE <60 days postvaccination against XBB sublineages was 74% (95% CI, 49%–87%) for hospitalization and 59% (95% CI, 48%–68%) for ED/UC, whereas VE against JN.1 sublineages for the same 2 outcomes was 50% (95% CI, 15%–71%) and 52% (95% CI, 39%–61%), respectively. VE ≥60 days postvaccination could not be calculated for XBB-related hospitalization due to small sample size, but was 39% (95% CI, 10%–59%) against XBB-related ED/UC visits 60–128 days after vaccination. VE was 57% (95% CI, 30%–73%) and 34% (95% CI, 22%–44%) against JN.1-related hospitalization and ED/UC visits 60–156 days after vaccination, respectively (Figure 2).

## DISCUSSION

In this test-negative case-control study, BNT162b2 XBB VE point estimates were 55%–65% against XBB-related outcomes but appeared lower against JN.1 sublineages (41%–54%), albeit with overlapping CIs. These findings persisted after accounting for time since vaccination. Consistent with prior reports [8, 9], VE was highest (>75%) against XBB-related hospitalization within 2 months of vaccine receipt, a period when XBB1.5-adapted vaccines were most closely matched to circulating XBB strains [1]. Our observational design has limitations that have been previously described [7, 10, 11], and it is possible that we misclassified SARS-CoV-2 sublineages in instances where we relied on SGTF or variant periods. Despite these potential limitations, our results—combined with reports of reduced neutralization activity against JN.1 [2, 3] and other early VE reports [4–6]—suggest that XBB vaccines likely have reduced effectiveness against JN.1 sublineages, which have become predominant globally. Thus, consistent with recent recommendations from the US Food and Drug Administration [12], the European Medicines Agency [13], and the World Health Organization [14], a vaccine strain change to target JN.1 sublineages, including KP.2, for the upcoming 2024–2025 season appears warranted.

## Supplementary Material

ofae370_Supplementary_Data

## Supplementary Data

Supplementary materials are available at *Open Forum Infectious Diseases* online. Consisting of data provided by the authors to benefit the reader, the posted materials are not copyedited and are the sole responsibility of the authors, so questions or comments should be addressed to the corresponding author.

## Notes

***Acknowledgments.*** T. B. F. and J. M. S. had full access to all of the data in the study and take responsibility for the integrity of the data and the accuracy of the data analysis. The authors would like to acknowledge Vennis Hong, MPH; Fagen Xie, PhD; Harpreet Takhar, MPH; and Sarah Simmons, MPH from the Kaiser Permanente Southern California (KPSC) Department of Research and Evaluation, who received support for their effort paid directly to KPSC. We also thank Joann M. Zamparo, MPH, who is an employee of Pfizer Inc and did not receive compensation beyond her salary, for her support of this project.

***Data availability.*** Anonymized data that support the findings of this study may be made available from the investigative team in the following conditions: (1) agreement to collaborate with the study team on all publications, (2) provision of external funding for administrative and investigator time necessary for this collaboration, (3) demonstration that the external investigative team is qualified and has documented evidence of training for human subjects protections, and (4) agreement to abide by the terms outlined in data use agreements between institutions.

***Disclaimer.*** The study design was developed by KPSC but approved by Pfizer. KPSC collected and analyzed the data; Pfizer did not participate in the collection or analysis of data. KPSC and Pfizer participated in the interpretation of data, in the writing of the report, and in the decision to submit the manuscript for publication.

***Financial support*.** This study was sponsored by Pfizer Inc.
